# The eutrophic rhytidoplasty: Subdermal tunneling and minimal skin undermining

**DOI:** 10.1007/s00238-012-0767-2

**Published:** 2012-10-12

**Authors:** Marcelo Daher, Alan Rodriguez Muñiz

**Affiliations:** Department of Plastic Surgery, Interclinica-Centroplastica, Rua Jardim Botânico 164, Jardim Botânico, Rio de Janeiro Brazil

**Keywords:** Eutrophic rhytidoplasty, Rhytidectomy, Rhytidoplasty, Facelift, Minimal skin undermining, Tunnelized skin detachment

## Abstract

**Background:**

Patients submitted to rhytidectomy usually relay their desire for briefer surgical procedures and a shorter post-operative period with faster recovery. In view of this, a technique in which the skin of the cervico-mandibular region is not undermined but only detached by blunt dissection was developed.

**Methods:**

A retrospective study of the senior author’s experience in eutrophic rhytidoplasty (EUR), the technique proposed herein, for facial rejuvenation was conducted. Patients submitted to EUR over a 10-year period were included in this study. A total of 224 interventions were performed. The procedure consists of using subdermal tunneling, performed with a cannula along the neck region and the midface area (combined or not with liposuction in certain areas, as needed), thus preserving nervous and vascular connections. The conventional skin undermining is minimal, just 4 cm around the auricular pavilion. Data were collected from the patients’ medical records at Interclínica—Centroplástica Clinic in Rio de Janeiro, Brazil.

**Results:**

Due to the skin eutrophic conditions, the operated patients, using the EUR technique, presented promising esthetical results. The mean rate of complications associated with the proposed technique was 1.7 %. The author found that this technique offers very promising results, a fact evidenced by the lower rate of complications among patients submitted to it.

**Conclusions:**

The EUR is a reliable option for facial rejuvenation due to the fact that it is less invasive, with a low morbidity and rate of complications. Its surgical time is reduced by 50 %, it has a shorter down-time, and yields natural results.

Level of Evidence: Level IV, therapeutic study.

## Introduction

There have been many techniques described to address facial aging. The history of rhytidoplasty reports in its literature the routine use of extensive dissections, as well as less invasive procedures with or without repositioning of the superficial muscular aponeurotic system (SMAS), liposuction, and lipofilling [1–3].

We have been performing facial rhytidoplasties since 1978 and have gone through several stages: initially, we performed rhytidoplasty using superficial skin traction, followed by the use of plicature, lipectomy of the cervico-mandibular region (CMR) in certain areas with fat deposits, and finally, treatment of the SMAS through undermining and traction [4–7]. It has been observed that conventional rhytidoplasty does bring back a youthful look. However, the skin develops a devitalized aspect as a result of the injury to several nervous and vascular connections occurring during extensive undermining. In fact, a less extensive undermining technique associated with the use of a blunt instrument, to help cutaneous detachment, can be safer and does yield better results [8–10], with less injury to the facial soft tissue while, at the same time, maintaining skin vitality [11–13].

With the demands of less invasive procedures building up, we have devised the “eutrophic” rhytidoplasty (from the Greek, *eu* = *well* and *trophein* = *to nourish*); thus, eutrophic means *“well nourished”*, a most appropriate term to define this procedure, characterized by being less invasive but without losing its wide reach.

In the past 10 years, the above procedure has been routinely performed, consisting of the detachment of the skin by subdermal tunneling of the face and neck with the help of a cannula and minimal conventional undermining of the skin around the auricular pavilion. The goal of this article is to describe the technical details of this conduct and show its benefits as well as its medium- and long-term results.

## Patients and methods

The experience and results achieved by a single surgeon using this technique were analyzed. In this analysis, 224 patients who received surgical treatment for facial aging were evaluated. All of the patients were operated on by the same surgeon, from July 2001 to July 2011, at the plastic surgery clinic Interclinica—Centroplastica located in Rio de Janeiro, Brazil. Data were collected from the patients’ medical records.

In the last 10 years, 224 patients of ages between 35 and 81 years were operated and evaluated. From this total, 86 % were females. In terms of ethnic groups, 77 % were Caucasians, 17 % were Mestizos, and the other 6 % were Negroes. Additionally, 14 % of the operated patients were either smokers or ex-smokers (Table 1).

**Table 1:** Socio-demographic data of evaluated patients

Demographic data		Eutrophic rhytidoplasty (number of patients)
Gender	Female	192 (86 %)
	Male	32 (14 %)
Ethnic group	Caucasian	172 (77 %)
	Mestizo	39 (17 %)
	Negroes	13 (6 %)
Smoking	Smoker	31 (14 %)
	Non-smoker	193 (86 %)

### Surgical technique

**Fig. 1:**
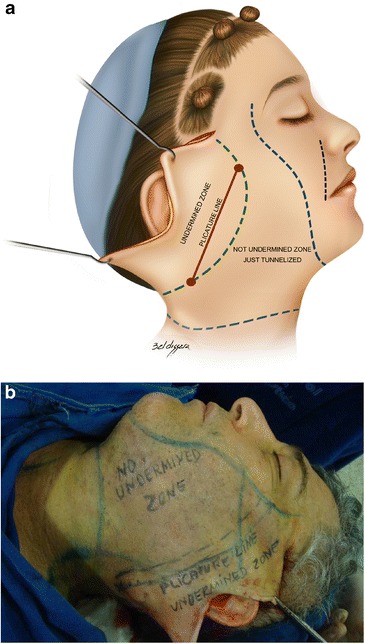
**a** EUR facial landmarks. *Black dotted* *line* nasogenian fold. *Blue dotted* *line* limit of tunneling with cannula. *Green dotted* *line* limit of the undermining with scissors. *Red line* plicature lina. **b** Trans-operative landmarks. All compromised regions in the demarcated areas show the tunneling with a cannula

**Fig. 2:**
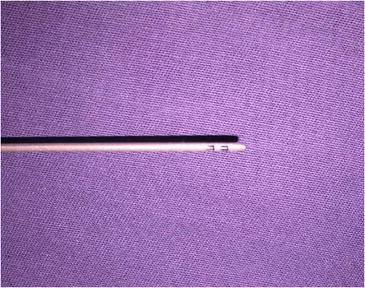
Flat tip cannula, 2-mm diameter (“shark head”)

**Fig. 3:**
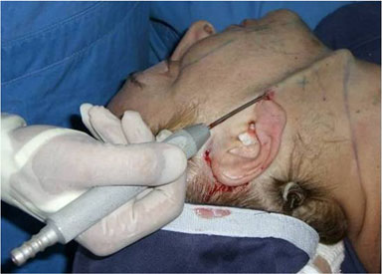
Aspects of subdermal tunneling performed with a cannula in the cervico-mandibular region

**Fig. 4:**
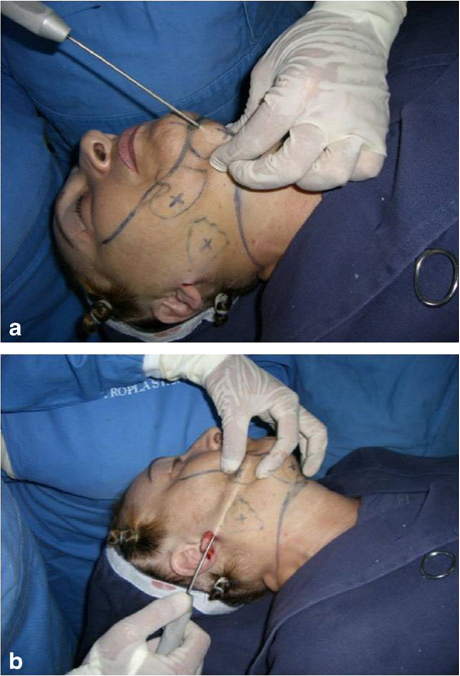
**a** Submandibular sculpting through submental liposuction of fat deposits, done before subdermal tunneling. **b** Subdermal tunneling up to the mandibular line. Observe the fat deposit areas already sculpted

**Fig. 5:**
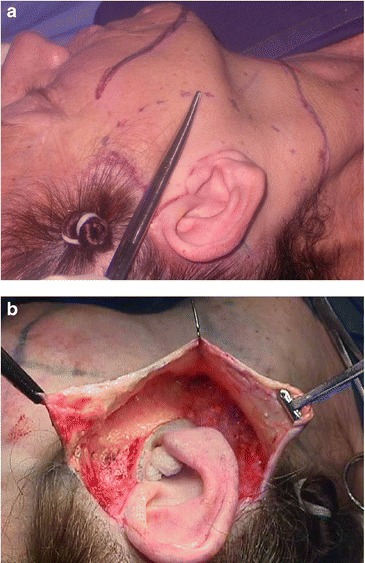
**a**, **b** Area of approximately 4 cm around the auricular pavilion undermined with scissors to allow SMAS plicature and excess skin resection

**Fig. 6:**
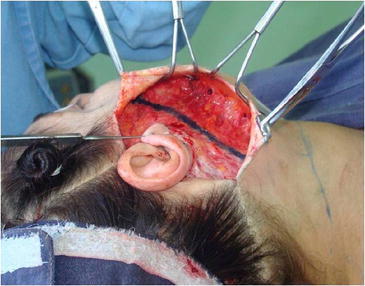
Details of SMAS plicature. Plication is parallel to the nasogenian fold or slightly divergent in the cervical region if the surgeon desires to emphasize the vertical traction using separated inverted stitches with nylon 3-0

**Fig. 7:**
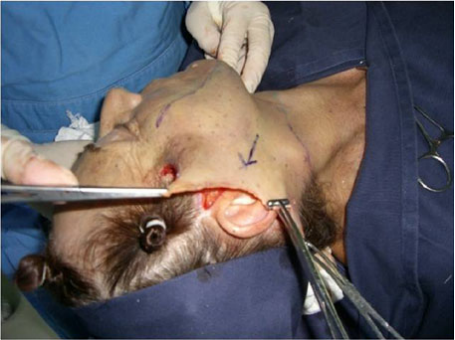
Traction of excess skin in a posterosuperior direction

Surgery is performed with the patient sedated and under local anesthesia. A bilateral demarcation is made to set the limits of the areas involved, from the malar region to the hyoid bone and the retroauricular region (Fig. 1a, b). The infiltration is made with lidocaine 0.125 % and adrenaline at 1.200.000 IU. All regions within the demarcation are infiltrated and then tunnelized with a “shark head”-type cannula (Fig. 2), with or without concomitant liposuction (depending on the presence of fat deposit areas) [8, 14] (Figs. 3, 4a and b). The conventional undermining of the skin, done with scissors, is performed from a pre-tragal incision, covering an area with a radius of approximately 4 cm around the auricular pavilion [9] (Fig. 5a, b). The SMAS plicature is sutured with separate stitches, keeping the knots inverted and using Nylon 3-0. It is done parallel to the nasolabial fold (NLF), starting from the lateral segment of the malar prominence, passing close to the earlobe, and reaching up to the cervical region. In some cases, when a more vertical traction is desired, the plicature line is divergent from the NLF caudally in the cervical region (Fig. 6). Then, traction of the excess skin is performed in the direction of the force vectors, one in a predominantly cephalic direction and another in a posterosuperior direction following the tragus-Darwin’s tubercle line, as described by Pitanguy [15], without counteracting the direction of the SMAS traction and the natural tendency that is more adequate to each face (Fig. 7). After blockage of the cutaneous flap with two stitches using Nylon 4-0 on the preauricular and retroauricular areas, the excess skin is resected without tension and skin synthesis is done with intradermal sutures using Monocryl 4-0 (Ethicon GmbH, Johnson & Johnson, Rio de Janeiro, Brazil) [16]. When required, auto-fat-grafts are made in the malar region, lips, and angle of the jaw [17]. An occlusive dressing is applied in the initial 8 h of the post-operative period, the amount of time during which the patient remains hospitalized.

## Results

A very fast recovery was achieved in the post-operative period, although the convalescence process was no different from that of a usual rhytidoplasty intervention. However, the technique proposed herein showed superior esthetic results, regarding the post-lifting skin texture, because this procedure opens itself to a normal and eutrophic appearance to the skin. The patients who underwent this surgery were satisfied with the results. They were all able to resume the same level of normal physical activity experienced before the surgery.

There were no significant complications in this series, and all of the patients developed prompt and prolonged esthetical results. There were no events that prolonged the usual post-operative stay. All of them had a great response to conservative management.

Only a few cases of small retroauricular hematoma were observed, without clinical significance. Regarding complications, we have documented one case of slight pre- and post-auricular skin loss close to the incision border in a patient who was a heavy smoker and one case of unilateral mandibular nerve paresis that was resolved in 6 months. No edema, seroma, infection, or other complications were encountered in this series (Table 2).

**Table 2:** Complications associated to surgical procedure

Complication	Eutrophic rhytidoplasty (224) (number of patients)
Alopecia	0
Edema^a^	0
Infection	0
Hematoma^b^	0
Hypertrophic scars	2 (0.9 %)
Seroma	0
Minimal skin loss^c^	0
Superficial cutaneous necrosis	1 (0.4 %)
Massive skin loss^d^	0
Mobility disorders^e^	1 (0.4 %)
Total	4 (1.7 %)

^a^Severe and prolonged edema

^b^Major hematoma as a primary clinical condition requiring surgical treatment

^c^Small skin losses close to the incision border

^d^Massive skin losses close to the incision border

^e^Marginal mandibular nerve pareses

The author does not consider ecchymosis as a complication, but as a minor condition. However, in past literature, several authors considered it as a complication [18].

The total rate of complications associated with the technique was only 1.7 % (four cases). The patients returned for long-term follow-up examination. As per the documentation, no patient expressed concern about the diagnosis, treatment, and expected outcomes during the follow-up.

The duration of the surgical procedure was significantly shortened (approximately 50 % of the usual time). Within a few hours of post-operative time, in a day-clinic regime, patients were in optimal conditions to be discharged.

## Discussion

The EUR as well as the conventional techniques demonstrated that they are efficient in the improvement of facial contour. Nevertheless, the use of a less invasive procedure can reduce the complications associated with the degree of undermining and likewise abbreviate the surgical time and the downtime [11, 19, 20]. The good results achieved in our experience are becoming more and more evident and are compensating our efforts at enhancements of our patients [21]. There is no doubt that a less invasive lifting can be performed successfully in facial rejuvenation, [22, 23] one that offers better overall results, specifically allowing the desired effect in a shorter recovery period and also a reduction in patient discomfort [1, 21, 24].

A similar finding has been recently described by Vicari and associates in a lipoabdominoplasty technique monitored with infrared thermography in which vascular patterns were compared, attesting the benefits of the method. The study evaluated abdominal skin vascularization and function, recognizing that a less invasive technique contributes to the preservation of skin circulation and to its thermoregulatory function with a lower complication rate [25]. The use of this concept in face lifting can provide a higher possibility of success, noting that it allows for a more natural repositioning of the tissue and structures that undergo anatomic migration as a result of the normal aging process [26, 27].

**Fig. 8:**
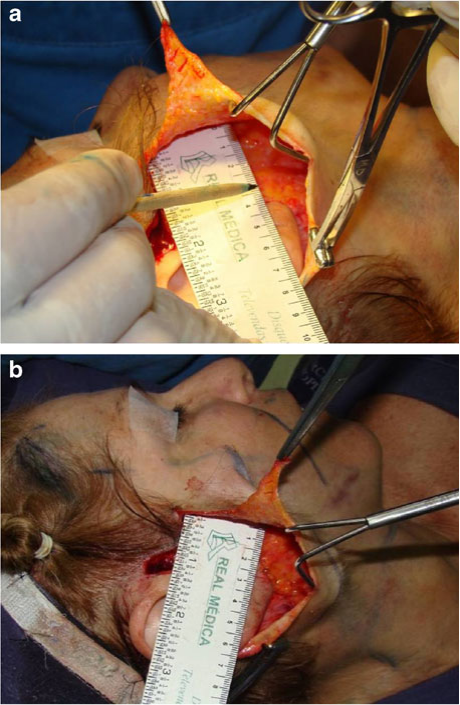
**a** Minimal skin undermining performed with scissors approximately 4 cm around the auricular pavilion. **b** After plication, the undermined area is reduced from 4 to 2 cm

In fact, the main purpose of this technique is to limit conventional skin dissection within a radius of 4 cm (Fig. 8a) from the external ear implantation, preserving skin quality, something that is not observed in traditional rhytidoplasties [28, 29]. This restricted undermined area provides sufficient space for the performance of SMAS plicature. After the plication, the remaining undermined area is reduced from 4 to 2 cm (Fig. 8b), thus minimizing dead space and eliminating the need to use drains.

**Fig. 9:**
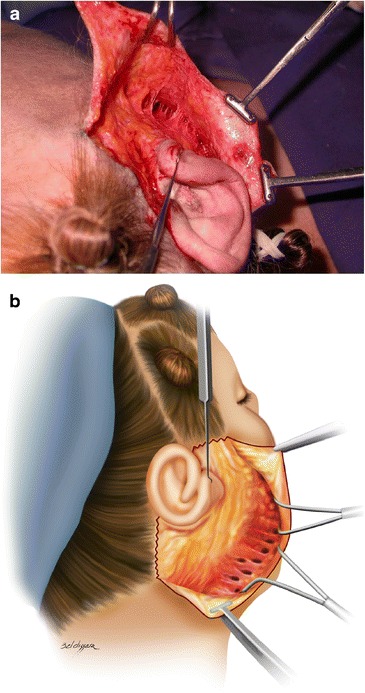
**a** Image showing the lifted skin exposing the trabeculae and blood vessels. **b** Tunnels and trabeculae aspects after tunneling

The trabeculae remaining after subdermal tunneling show the integrity of vascular connections (Fig. 9a, b), with the skin presenting extensive mobilization to traction similar to that observed in more extensive dissections [20, 30–32].

**Fig. 10:**
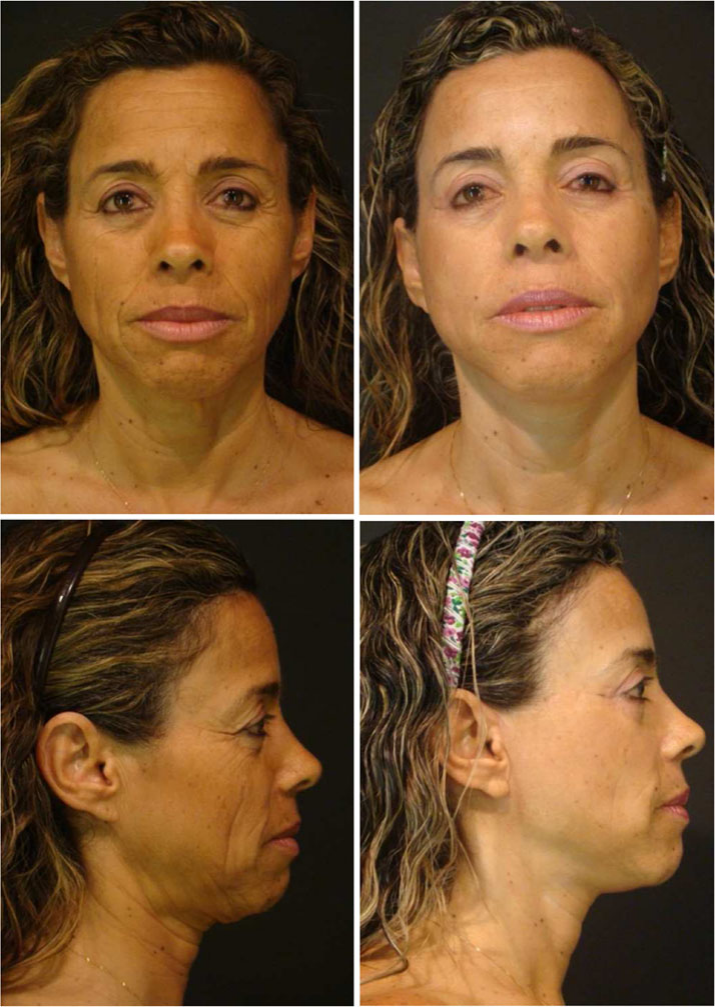
Left pre-operative photographs of a 48 years old woman. Right post-operative photographs taken 9 months after the patient underwent to EUR. In two basic postures: straight on and profile. Blepharoplasty and malar lipofilling were performed

**Fig. 11:**
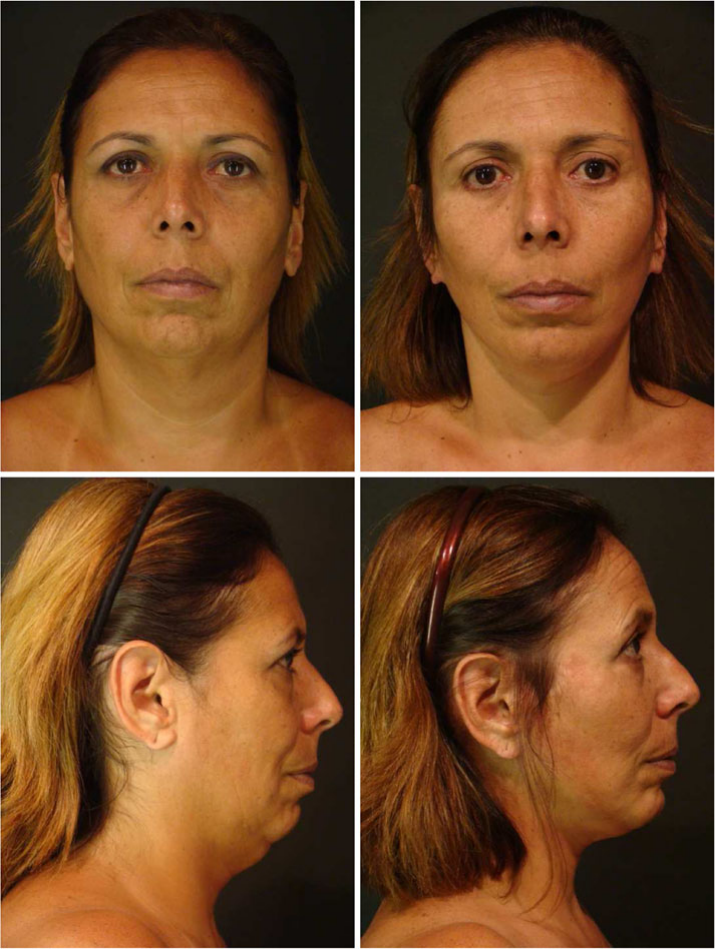
Left preoperative photographs of a 46 years old woman. Right late postoperative photographs taken 36 months after the patient underwent to EUR, in frontal and lateral views. Blepharoplasty, liposuction of fat deposits areas, lipofilling of malar area, mandibular angle and lips were associated

Due to the characteristics of this procedure, the submentonian area can also be treated. The senior author has systematized a face lift technique where the facial skin is not undermined in the CMR region, just tunnelized with a cannula in the subdermal plane. The platiysmal bands, medially, are treated by making an incision of approximately 5 cm above the submentonean crease where an undermining, just enough to expose the area without affecting the principles of the technique, is performed. Also, it depends on the surgeon’s preference.

Liposuction of general and localized adipose tissue on the face and neck is performed [33, 34] while maintaining the principles of facial volume. The reimplantation of the aspirated fat has its specific indication to address esthetic deficiency for the following anatomical regions: the nasolabial folds, malar region, mandible (jawline), and lips [27, 35–37].

With the reduction of the dissected area, drainage becomes unnecessary and the incidence of hematomas decreases to near zero in all of the operations using the EUR technique [9, 21]. In the post-operative period, edema and ecchymosis are also reduced, therefore permitting the patient to have an early return to normal daily activities and social life.

The recovery period for returning to sports activities and sun exposure remains at 3 months. Surgical procedures such as blepharoplasty, rhinoplasty, and treatment of platysmal bands through submandibular incision may be combined due to the low morbidity of the technique and the reduced surgical time in approximately 50 % as compared to that of conventional rhytidoplasty.

Moreover, the proposed technique could be performed in almost all cases, although it may be the preferred procedure for a selected group of patients. For routine cases, this procedure can be helpful in the elderly patient, with or without associated medical comorbidity (diabetes, hypertension, obesity), who is able to withstand surgery and local anesthesia. The intervention provides many benefits in the surgical care of this kind of patient.

This approach is useful, particularly in situations with clinical suspicion of poor cutaneous blood perfusion as in the case of heavy-smoker patients. Because EUR is a less aggressive and also a faster surgical procedure, those patients have minimal inconvenience with this approach. Outcomes achieved with this procedure are comparable to or better than those obtained with standard surgical ones. However, not all patients are eligible, so it is certain that to obtain the desired result, in other cases it would be necessary to be more aggressive.

Finally, the so-called stigmatized face (shiny and stretched) that sometimes resulted from a conventional rhytidoplasty due to extensive dissection and excessive traction was not observed in the cases submitted to the presented technique [38–40] (Figs. 10 and 11).

## Conclusion

Eutrophic rhytidoplasty has offered natural results, higher safety levels in the handling of skin layers, less post-operative recovery time and, performed by us, a proven reduction of 50 % in surgical time.

The success rate is due to the less invasive nature of the technique and the very promising prospective result based on its eutrophic effect. This technique does not require the use of drains or dressings, noting that it presents a low incidence of bleeding and reduced dead spaces. In addition, its setback as well as complication rate is near zero. These positive aspects are emphasized by the results achieved in our cases throughout the last 10 years.

## References

[1] Ramirez OM (2001). Full face rejuvenation in three dimensions: a “face-lifting” for the new millennium. Aesthetic Plast Surg.

[2] Brackup AB (2003) Advances and controversies in face lift surgery. Curr Opin Ophthalmol 14(5):253–259 10.1097/00055735-200310000-00005 14502052

[3] Warren RJ, Aston SJ, Mendelson BC (2011). Face Lift. Plast Reconstr Surg.

[4] Adamson PA, Litner JA (2005). Evolution of rhytidectomy techniques. Facial Plast Surg Clin North Am.

[5] Aston SJ (1983). Platysma—SMAS cervicofacial rhytidoplasty. Clin Plast Surg.

[6] Baker D (2000). Rhytidectomy with lateral SMASectomy. Facial Plast Surg.

[7] De Castro Cardoso C, Aboudib JH (1980). Extensive cervical and lower face lipectomy: its importance and anatomical basis. Ann Plast Surg.

[8] Teimourian B (1983). Face and neck suction-assisted lipectomy associated with rhytidectomy. Plast Reconstr Surg.

[9] Stocchero IN (2007). Shortscar face-lift with the RoundBlock SMAS treatment: a younger face for all. Aesthetic Plast Surg.

[10] Gryskiewicz JM (2003) Submental suction-assisted lipectomy without platysmaplasty: pushing the (skin) envelope to avoid a face lift for unsuitable candidates. Plast Reconstr Surg 112(5):1393–1405; discussion 1406–1407 10.1097/01.PRS.0000083222.41089.DD 14504526

[11] Cárdenas-Camarena L, Encinas-Brambila J, Guerrero MT (2011). Cervicofacial rhytidoplasty: more does not mean better. Aesthetic Plast Surg.

[12] Saldanha OR, de Azevedo SFD (2010). Ritidoplastia com descolamento composto/Facelift with composite undermining. Rev Bras Cir Plást.

[13] Hamra ST (1984). The tri-plane face lift dissection. Ann Plast Surg.

[14] Daher JC, Cosac OM, Domingues S (1988). Face-lift: the importance of redefining facial contours through facial liposuction. Ann Plast Surg.

[15] Pitanguy I (2000). The round-lifting technique. Facial Plast Surg.

[16] Rees TD, Wood-Smith D (1973). Cosmetic facial surgery.

[17] Sandoval SE, Cox JA (2009). Facial fat compartments: a guide to filler placement. Semin Plast Surg.

[18] Gladstone GJ, Myint S, Black EH, Brazzo BG, Nesi FA (2005). Fundamentals of facelift surgery. Ophthalmol Clin North Am.

[19] Del Campo AF (2008). Update on minimally invasive face lift technique. Aesthet Surg J.

[20] Myckatyn TM, Mackinnon SE (2004). A review of facial nerve anatomy. Semin Plast Surg.

[21] Litner JA, Adamson PA (2006). Limited vs extended face-lift techniques: objective analysis of intraoperative results. Arch Facial Plast Surg.

[22] Rohrich RJ, Taylor NS, Ahmad J, Lu A, Pessa JE (2011). Great auricular nerve injury, the “subauricular band” phenomenon, and the periauricular adipose compartments. Plast Reconstr Surg.

[23] Ellsworth WA, Basu CB, Iverson RE (2009). Perioperative considerations for patient safety during cosmetic surgery—preventing complications. Can J Plast Surg.

[24] O’Connell JB (2003). Refinements of minimal-incision rhytidectomy. Eur J Plast Surg.

[25] Vicari Nogueira CHF. How does thermography helps abdominal plastic surgery. International Consensus and Guidelines on Medical Thermology. Thermology International 20/4 2010

[26] Graf R, Groth AK, Pace D, Neto LG (2008). Facial rejuvenation with SMASectomy and FAME using vertical vectors. Aesthetic Plast Surg.

[27] Rohrich RJ, Pessa JE (2007). The fat compartments of the face: anatomy and clinical implications for cosmetic surgery. Plast Reconstr Surg.

[28] Hudson DA (2010). An analysis of unsolved problems of face-lift procedures. Ann Plast Surg.

[29] Saylan Z (1999). The S-lift: less is more. Aesthet Surg J.

[30] Da Luz DF, Wolfenson M, Figueiredo J, Didier JC (2005). Full-face undermining using progressive dilators. Aesthetic Plast Surg.

[31] Jones BM, Grover R (2004). Reducing complications in cervicofacial rhytidectomy by tumescent infiltration: a comparative trial evaluating 678 consecutive face lifts. Plast Reconstr Surg.

[32] Massiha H (2003). Short-scar face lift with extended SMAS platysma dissection and lifting and limited skin undermining. Plast Reconstr Surg.

[33] Conell BF (1981). Surgical technique of cervical lift and facial lipectomy. Aesthetic Plast Surg.

[34] Avelar J (1985). Fat-suction of the submental and submandibular regions. Aesthetic Plast Surg.

[35] Donofrio LM (2000). Fat distribution: a morphologic study of the aging face. Dermatol Surg.

[36] Tzikas TL (2004). Lipografting: autologous fat grafting for total facial rejuvenation. Facial Plast Surg.

[37] Rohrich RJ, Ghavami A, Lemmon JA, Brown SA (2009). The individualized component face lift: developing a systematic approach to facial rejuvenation. Plast Reconstr Surg.

[38] Sullivan CA, Masin J, Maniglia AJ, Stepnick DW (1999). Complications of rhytidectomy in an otolaryngology training program. Laryngoscope.

[39] Rodriguez-Bruno K, Papel ID (2011). Rhytidectomy: principles and practice emphasizing safety. Facial Plast Surg.

[40] Gloster HM (2008). Complications in cutaneous surgery, chapter 19.

